# Abortion experiences among Zanzibari women: a chain-referral sampling study

**DOI:** 10.1186/s12978-016-0129-9

**Published:** 2016-03-11

**Authors:** Alison Norris, Bryna J. Harrington, Daniel Grossman, Maryam Hemed, Michelle J. Hindin

**Affiliations:** Department of Population, Family and Reproductive Health, Johns Hopkins Bloomberg School of Public Health, 615 North Wolfe Street, Baltimore, MD 21205 USA; Present address: College of Public Health, The Ohio State University, 326 Cunz Hall, 1841 Neil Ave, Columbus, OH 43210-1351 USA; Yale College Charles P. Howland Fellow, 74 High Street, New Haven, CT 06511 USA; Present address: Department of Epidemiology, Gillings School of Global Public Health, University of North Carolina at Chapel Hill, 2101 McGavran-Greenberg Hall CB#7435, Chapel Hill, NC 27599 USA; Ibis Reproductive Health, 1330 Broadway, Suite 1100, Oakland, CA 94612 USA; Bixby Center for Global Reproductive Health, Department of Obstetrics, Gynecology and Reproductive Sciences, University of California, San Francisco, CA USA; African Union Commission, Department of Medical Services, Addis Ababa, Ethiopia

**Keywords:** Zanzibar, Abortion, Pregnancy, Reproductive health, Post abortion care, Chain-referral sampling

## Abstract

**Background:**

In Zanzibar, a semi-autonomous region of Tanzania, induced abortion is illegal but common, and fewer than 12 % of married reproductive-aged women use modern contraception. As part of a multi-method study about contraception and consequences of unwanted pregnancies, the objective of this study was to understand the experiences of Zanzibari women who terminated pregnancies.

**Methods:**

The cross-sectional study was set in Zanzibar, Tanzania. Participants were a community-based sample of women who had terminated pregnancies. We carried out semi-structured interviews with 45 women recruited via chain-referral sampling. We report the characteristics of women who have had abortions, the reasons they had abortions, and the methods used to terminate their pregnancies.

**Results:**

Women in Zanzibar terminate pregnancies that are unwanted for a range of reasons, at various points in their reproductive lives, and using multiple methods. While clinical methods were most effective, nearly half of our participants successfully terminated a pregnancy using non-clinical methods and very few had complications requiring post abortion care (PAC).

**Conclusions:**

Even in settings where abortion is illegal, some women experience illegal abortions without adverse health consequences, what we might call ‘safer’ unsafe abortions; these kinds of abortion experiences can be missed in studies about abortion conducted among women seeking PAC in hospitals.

## Background

Unsafe abortion remains a significant cause of morbidity and mortality globally [1, 2], and the stigmatized nature of abortion makes it challenging to research [3]. Many hospitals, both in settings where abortion is legal and illegal, provide post abortion care (PAC) services. The aim of PAC is to reduce deaths and short and long term complications from incomplete and or unsafe abortions. It includes emergency treatment for complications of spontaneous or induced abortion – typically with manual vacuum aspiration (MVA) or dilation and curettage (D&C) – as well as family planning counseling and provision, and counseling and testing for sexually transmitted infections. Research about illegal abortion is often conducted among women who come to hospitals for PAC, including women with incomplete abortions or with abortions that were in some way worrisome to the women. Hospital-based studies of abortion may provide an incomplete picture of abortion for a particular community, because many women complete an illegal abortion without complications and without PAC (so these ‘safer’ illegal abortions go unrecognized), while others who need medical care after unsafe abortion, may not or cannot seek PAC (and these unsafe illegal abortions also would go unrecognized) [4].

In Tanzania, abortion is widely practiced, and most abortions are unsafe, because abortion is only legal if the pregnancy is a threat to the woman’s life [5]. Most abortion research in east Africa is conducted among patients receiving facility-based PAC [6, 7]. Up to 60 % of women admitted to Tanzanian hospitals with ‘miscarriage’ have had an induced abortion [6, 8, 9]. Unsafe abortion accounts for an estimated 17–21 % of maternal mortality in Tanzania [10].

In Zanzibar, a semi-autonomous region of Tanzania, fewer than 12 % of married reproductive-aged women use modern contraception (such as injectables, birth control pills, intrauterine devices, implants, or condoms) [11] and unintended pregnancy is correspondingly common. From studies on mainland Tanzania, we know that women use a wide variety of methods to terminate unwanted pregnancies, including ingesting (or inserting into the vagina) a variety of locally grown herbs, chloroquine, laundry detergent, and ashes [12, 13]. Complications in these settings include infection and fever, heavy vaginal bleeding, and cervical or uterine trauma [14]. Despite the public health significance of abortion, and the availability of published studies about abortion on mainland Tanzania, there are no studies about abortion in Zanzibar in the peer-reviewed literature. Zanzibar represents an interesting case for several reasons: first, despite its cosmopolitan placement in the Indian Ocean world, the archipelago has extremely low contraceptive prevalence rate (12 % among married women) relative to mainland Tanzania (28 % among married women) [11] and the rest of east Africa. Next, Zanzibar differs from the mainland in other significant ways: the population is 95 % Muslim, most residents live relatively close to tertiary care (because the islands are small), and most people, even rural dwellers, have rich social networks throughout the islands. Finally, in 2008, members of the Zanzibar Ministry of Health noted that the female gynecological ward was extremely busy with PAC services, and were keen to understand why. Motivated by these factors, we conducted a multi-methodological study of contraception and the consequences of unwanted pregnancy in Zanzibar. In this manuscript, we provide an analysis of data about the termination experiences of a community-based sample of Zanzibari women. Using the results of 45 interviews with women recruited via chain-referral sampling, we report the characteristics of women who have had abortions, the reasons they had abortions, and the methods they used to terminate their pregnancies.

## Methods

### Recruitment

Women commonly fail to self-report abortion in interviews because of the stigma surrounding abortion [3]. Thus, we used a network-based sampling method known as chain-referral or snowball sampling to recruit women from the urban and peri-urban communities surrounding Zanzibar town who had terminated a past pregnancy. Chain-referral sampling is a non-probability sampling technique that is particularly useful to reach ‘hidden’ populations with stigmatized or illegal behaviors because participants are recruited by acquaintances who themselves participated in the research [15, 16]. With the chain-referral method, the sample is initiated by the research team recruiting some known members of the target population (the ‘seeds’). These seeds then recruit a small number of others in the population (who become the first ‘wave’). Members of each wave in turn recruit other participants.

**Fig. 1:**
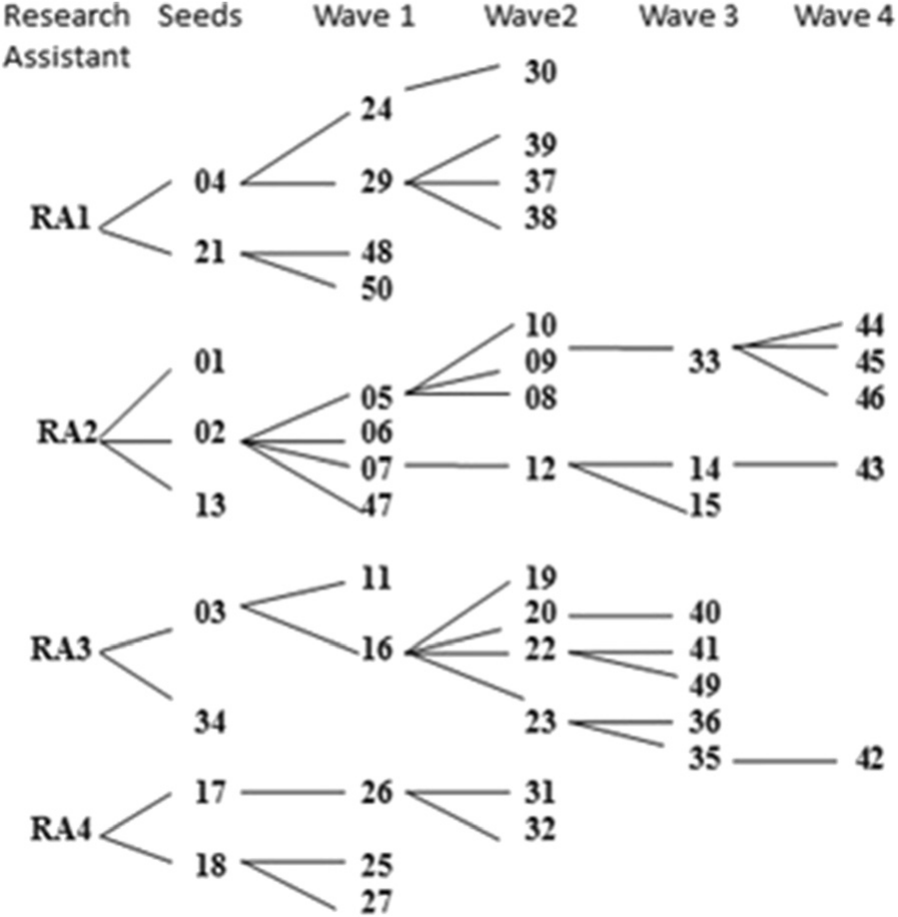
Chain-referral sampling recruitment tree: Four research assistants (RA1-RA4) invited nine women who had had an abortion to be the “seed” participants. After participating, these seeds in turn invited women who had had an abortion to participate. Each wave represents one referral removed from the seed

Members of our research team (represented in Fig. 1 by “RA”) invited acquaintances they knew had had abortions from prior personal conversations to be interviewed. Those who agreed to be interviewed were the nine seeds. Upon conclusion of their interview, each of the seeds, and then all participants up to the 40^th^ participant (at which point new recruitment was closed), were asked if they were interested in inviting others to participate. Those who were willing were given recruitment cards to share with up to three other women known to them who had had an induced abortion. The cards indicated that we were researchers interested in talking with women about their experiences and thoughts about reproductive health, and included a number for women to call for more information. In practice, all recruited women were brought directly to the interview site by the acquaintance who recruited them. The recruitment continued in this way extremely rapidly between May 15 and May 31, 2010, for five waves. Of the 49 women we interviewed, 45 had had one or more induced abortions. Our participants recruited between zero and four new participants, with a mean of 1.0 (standard deviation 1.2).

### Participants and procedures

To be included, all participants had to be 15 years or older, be able to give informed consent, be able to participate in a Swahili language interview, be a resident of one of Zanzibar’s islands, and self-report an induced abortion. We explained an “induced” abortion was a pregnancy that was terminated due to something the woman did (or had done).

Research assistants invited acquaintances who they knew had terminated a past pregnancy to come to the office to learn more about what study participation involved. If they were willing to participate, verbal informed consent was obtained. Likewise, women who were recruited from a seed or a woman in one of the waves came to the research office, research assistants explained the purpose and content of the interview, then read the informed consent form prior to proceeding with the interview. All women who came to the office agreed to participate. Interviews were conducted in Swahili by Zanzibari research assistants trained in empathic interviewing techniques, following a semi-structured interview guide. The interviews took place in private rooms within our study office, which was located near the main market area in Zanzibar town. We obtained informed consent for audio-recording the interviews. For participants who declined audio-recording, interviewers wrote notes during interviews to record participants’ responses. Each interview lasted approximately one hour. All interview participants were given 3000 Tanzanian shillings (equivalent to US$2) for their time.

The interview included basic demographic questions, as well as questions about the details of participants’ induced abortion, age at abortion, relationship status at the time of the abortion, and reasons for termination. Each participant was asked about the steps she took and methods she used to terminate the pregnancy. Participants were asked whether they had visited a hospital for care following the induced abortion. The interviewers asked probing follow-up questions to elicit details, but participants were not prompted with lists.

### Analysis

Responses were entered into spreadsheets for analysis. Qualitative data were hand coded for analytic categories; categorical information was tabulated, and frequencies were calculated. We present here a descriptive analysis of the data.

### Ethics, consent and permissions

This research was approved by the Johns Hopkins Bloomberg School of Public health Institutional Review Board on October 28, 2009 (IRB#00002375) and by the Zanzibar Medical Research Ethics Committee on December 31, 2009 (IRB#0019/09).

## Results

### Participant characteristics

We interviewed 49 women, but four were excluded from analysis because they reported a spontaneous abortion instead of induced abortion. Five other women reported more than one abortion; two of these women gave in-depth information on both abortions. In sum, we present information on 47 terminations from 45 women. We have no evidence that any of these women would have had grounds for a legal abortion.

All participants lived in or near to Zanzibar town, were Muslim and nearly all (91 %) had at least some secondary-level education (Table 1). Participants’ ages at the time of the interview ranged from 20 to 52 years (mean 31.1 years) and participants had a minimum of one lifetime pregnancy (*n* = 10, 22 %) and a maximum of 8 pregnancies, with half of the women reporting four or more pregnancies (*n* = 23, 51 %), giving an overall mean of 3.5 pregnancies per participant (Table 1). At the time of the interview, participants had between zero and six living children (mean 2.1), with half of the women having 1 to 3 children. Women reported a range of time since pregnancy termination of 0 to 23 years (median 4.0 years).

**Table 1:** Participant characteristics at time of interview (*n* = 45 participants)

		*N*	(%)
Age at interview (mean 31.1 years)			
	<20 years	0	(0)
	20–29 years	24	(53)
	30–39 years	13	(29)
	>40 years	8	(18)
Relationship status at interview			
	Single, never married	10	(22)
	Married	30	(67)
	Divorced/widowed	5	(11)
Number of lifetime terminations			
	1	40	(89)
	2	4	(9)
	3+	1	(2)
Number of pregnancies at time of interview			
	1	10	(22)
	2	8	(18)
	3	4	(9)
	4+	23	(51)
	Range (mean)	1–8	(3.5)
Number of living children at time of interview			
	0	11	(25)
	1	8	(18)
	2	8	(18)
	3	6	(14)
	4+	11	(25)
	Missing	1	
	Range (mean)	0–6	(2.1)
Ever contraception use (lifetime)			
	Never used contraception	7	(16)
	Calendar/rhythm method or withdrawal	12	(27)
	Condom	9	(20)
	Oral contraceptive pills	25	(57)
	Depo injection	15	(34)
	Hormonal implant or IUD	5	(11)
	Tubal ligation/hysterectomy	1	(2)

Participants reported varied abortion experiences and contexts. At the time of pregnancy termination, the youngest age reported was 13 and the oldest was 42 (mean 24.6 years) (Table 2). When they had their abortion, 43 % (*n* = 20) of participants were single and had never been married, 43 % (*n* = 20) were married, and 14 % (*n* = 7) were divorced or widowed. For over a third of participants, women terminated their first pregnancy (*n* = 17, 36 %), 19 % terminated the second (*n* = 9), and 15 % (*n* = 7) terminated the third pregnancy. More than a quarter of terminated pregnancies were the woman’s fourth pregnancy or higher (*n* = 14, 30 %). The gestational age of the terminated pregnancies (based on self-report) ranged from three weeks to four months (mean 1.8 months), with over three quarters of women reporting they terminated the pregnancy when it was between 1 and 3 months (Table 2).

**Table 2:** Participant characteristics at time of abortion (*n* = 47 abortions)

		*N*	(%)
Age at termination (mean 24.6 years)			
	< 20 years	11	(25)
	20–29 years	24	(55)
	30–39 years	8	(18)
	>40 years	1	(2)
	Missing	3	
Relationship status at termination			
	Single, never married	20	(43)
	Married	20	(43)
	Divorced/widowed	7	(14)
Primary reason(s) for termination ^a^			
	Had young child	16	(34)
	Extramarital pregnancy	16	(34)
	Was student in school	14	(30)
	Shame: societal, parental	11	(23)
	Financial concerns	10	(21)
	Relationship concerns	6	(13)
	Housing concerns	4	(9)
	Had many children	3	(6)
	Health concerns	3	(6)
	Didn’t want pregnancy at that time	2	(4)
Number of pregnancies at time of termination			
	1	17	(36)
	2	9	(19)
	3	7	(15)
	4+	14	(30)
	Range (mean)	1–8	(2.6)
Using contraception at time of pregnancy			
	Not using contraception	29	(62)
	Calendar/rhythm method or withdrawal	11	(23)
	Condom	3	(6)
	Oral contraceptive pills	3	(6)
	Depo injection	1	(2)
	Hormonal implant or IUD	0	
	Tubal ligation/hysterectomy	0	
Gestational age at termination (months)			
	< 1	2	(5)
	1 to < 2	15	(38)
	2 to < 3	15	(38)
	3 to < 4	6	(15)
	4+	1	(3)
	Missing	8	

^a^Multiple responses allowed; total > 100 %

### Reason for terminating pregnancy

In discussing their abortions, participants described conditions under which continuing an unwanted pregnancy would threaten a successful future. Many women responded with multiple reasons why they terminated their pregnancies. The two most common reasons cited were ‘having an infant’ (*n* = 16, 34 %) and ‘extramarital pregnancy’ (*n* = 16, 34 %). Those women who reported having an infant as a reason for termination were worried about their ability to cope with the responsibility of a second infant. Also, those with infants were concerned about the social expectation that they should breastfeed the current infant for two full years, conflicting with another social norm that they should stop breastfeeding as soon as a new pregnancy was known.

Conceiving the pregnancy outside of marriage, whether due to never having been married, being divorced, or conceiving with a man who was not a husband, was commonly cited as a reason to terminate a pregnancy (*n* = 16, 34 %). Shame of pregnancy was mentioned by many participants (*n* = 11, 23 %), including feeling ashamed of what the woman’s parents and other people would think about her having a pregnancy at that time. While pregnancy among never married women is more stigmatized than pregnancy among divorced women, divorced or widowed women said they terminated to avoid the shame of having a child out of wedlock. For one married woman, a pregnancy was shameful proof of sexual intercourse outside of her marriage.

Nearly a third (*n* = 14, 30 %) of participants explained that they decided to have an abortion because they were students at the time. For students, almost all unmarried, pregnancy reveals that they have had socially unsanctioned sexual intercourse. In addition to the social stigma, bearing a child may preclude successful completion of schooling.

Participants described concerns about financial well-being and/or housing, including an explanation that ‘*maisha ni magumu*’ (‘life is hard’), that the woman’s residence was not big enough for a new baby, or that the woman lived with her parents who would not welcome an infant. Women who were not married feared dissolution of their current romantic union and expulsion from their natal homes. For a few participants, health concerns (previous difficult labor, older maternal age) and relationship concerns (abusive husband, separated from partner) were reasons for terminating pregnancies.

### Methods of pregnancy termination

Most women (39 of 47, 83 %) were successful on the first termination attempt, which often involved a combination of methods (Table 3). The most effective first attempt was a termination at a hospital, either public or private (*n* = 15, 32 % of 1^st^ attempts). Some women initially received assistance from a ‘daktari’ (literally translates to *doctor*, but the term is widely used to connote any person who is associated with the health care system regardless of clinical credentials, and we have translated as ‘medical worker’) but not at a health care facility (*n* = 4, 9 %). Overall, 8 of the 47 abortions required a second attempt to terminate the pregnancies. Many participants described using ‘hospital’ or ‘medical worker’s house’ as the place for obtaining the abortion; ultimately half the sample had a successful abortion with a medical worker: 43 % (*n* = 20) in a hospital setting and 11 % (*n* = 5) in a medical worker’s house. Women paid between 0 and 70,000 Tanzanian Shillings ($0–47 USD) for a *daktari* to terminate their pregnancies, with an average payment of 43,250 Tanzanian Shillings ($29 USD).

**Table 3:** Methods participants used to abort pregnancies (*n* = 47)

	First method^a^		Final method^b^	
Method of termination	*N*	(%)	*N*	(%)
Hospital	15	(32)	20	(43)
Medical worker’s house	4	(9)	5	(11)
Henna root alone	5	(11)	5	(11)
Strong black tea alone	2	(4)	1	(2)
Tetracycline alone	1	(2)	1	(2)
Combo: Henna root, strong black tea	4	(9)	1	(2)
Combo: Henna root, strong black tea, traditional medicine^c^	3	(6)	3	(6)
Combo: Henna root, strong black tea, tetracycline	1	(2)	1	(2)
Combo: strong black tea, chloroquine	1	(2)	1	(2)
Combo: traditional medicine^c^, strong black tea	5	(11)	5	(11)
Traditional medicine^c^	6	(13)	4	(9)

^a^first method includes participants for whom this method succeeded or failed

^b^final method count excludes participants who used this method but were not successful with it; final method count includes participants who used this method successfully after failing a different first method

^c^including a variety of herb and plants, for example: *Plectranthus* spp, papaya tree root, cassava plant leaves, mango tree seeds, garlic, ground chalk, and lime tree root

In describing the methods used by providers to terminate their pregnancies, it was clear that many women did not understand the termination procedure. Some descriptions are recognizable as manual vacuum aspiration (MVA) (‘by suction’), and others as dilatation and curettage (D&C) (‘by something metal with a tip’). Other explanations are partial or vague: ‘by use of a speculum’ commonly referred to as '*domo la bata*' (duck’s beak), ‘by medication,’ ‘by a syringe with a blue solution,’ ‘with scissors,’ ‘by injection,’ and ‘by a straw.’ Some participants had many details without understanding how medication or devices were used: ‘I received pills, three small yellow ones, some longer oval pills, and something like tetracycline’, or ‘[the provider used] one tool which had an angle here and here of metal, like it had a point.’ Of the five women who terminated at a medical worker’s home, two described use of a speculum and a pointed instrument, one described MVA, one described medicine inserted into the vagina, and one described two visits to the provider: at the first, the provider used scissors, then after some time the woman returned and the provider removed the pregnancy – the method used was unclear but the woman used the same word typically used to describe MVA. The majority of the hospital terminations involved what is likely MVA or D&C; some also noted they received injections or other medications but none were specific enough to the identify the drugs used. Descriptions of providers themselves are similarly vague, usually referring to *daktari*. We do not know whether the *daktari* described by our participants were medical practitioners trained in PAC or people who were specially known to the clients. Finally, three participants reported going to a hospital for PAC after terminating their pregnancies: two for complications after self-induction efforts (for uterine pain or bleeding) and one for reassurance. The rest had successful abortions at home or in clinics without utilizing PAC. No participants had active symptoms suggesting an ongoing termination in need of PAC or medical attention at the time of interview.

While many participants engaged with members of the health care system, nearly half (*n* = 22, 47 %) successfully terminated a pregnancy using herbs. Nearly a quarter of participants specified using a drink made from the root of the henna plant (*Lawsonia inermis*), alone or in combination with black tea or other herbs (*n* = 10, 21 %). Henna is a known abortifacient [17]. Other reported methods used in combinations included drinking strong tea (made from boiling a kilogram of black tea leaves down to a single cup of tea) (*n* = 12, 26 %), taking other traditional medicine (*n* = 12, 26 %) and overdosing on medications (*n* = 3, 6 %). In the category of ‘traditional medicine,’ we captured a combination of plant products, including *Plectranthus* species, papaya tree root, cassava plant leaves, mango tree seeds, garlic, ground chalk, and lime tree root, some of which may be abortifacients.

## Discussion

### Main findings

Zanzibari women terminate pregnancies using multiple methods, for a range of reasons, at various life stages, and usually without complications requiring PAC. While abortions from medical providers were most often effective, nearly half our participants aborted without medical providers. The broad variation of experiences is noteworthy in light of our non-random sampling method.

Women in Zanzibar terminate pregnancies in a situation of multiple uncertainties. While some legal exceptions exist for abortion in Tanzania, a Center for Reproductive Rights report states that Tanzania’s law and policies about abortion are inconsistent and unclear [18]. Most women (and people) in Zanzibar believe that all abortion is illegal. And in the Zanzibari context, illegal abortion provision is not regulated with oversight from the health care system, and thus by definition, is unsafe. Nevertheless, women in our study described a range of abortion methods and experiences, most of which terminated the pregnancy without physical sequelae or need for PAC. It is possible that women who had a *daktari* terminate their pregnancies were given prophylactic antibiotics, thus reducing the risk of infection and potentially seeking formal PAC at the hospital; two participants mentioned being given pills by the *daktari* but they did not know what the pills were. A clear dichotomy between ‘safe’ and ‘unsafe’ may not represent reality in Zanzibar. While we cannot classify illegal abortions as ‘safe’ (because of the lack of regulatory oversight), those our participants experienced were perhaps ‘safer’ unsafe abortions. For example, using misoprostol or MVA for an early termination is illegal, but if used in recommended ways, it may be safe, even if the woman later presents for PAC for perceived incomplete abortion. While we do not know anything of the medical credentials of the *daktari* who provided terminations, they had some knowledge of abortion procedures, and some used what sounds like MVA or D&C techniques despite conducting terminations at non-health facility locations. Many of the abortion methods used by our participants have been reported in other studies in Tanzania [19]. The persistent use of these unregulated methods reflect the lack of easy alternative abortion options and signify the need for abortion policy which will pave the way for safe abortion services.

In this exploratory study, we demonstrate that chain-referral sampling is effective in gathering abortion experiences from a community-based population. In addition to efficiency (identifying people with characteristics of interest), participants came in with trust, having been recruited by someone they knew, and they were aware that participation involved talking about induced abortion, reducing likelihood of misclassification. Abortion is not rare among Zanzibari women, but it goes largely unreported, so chain-referral sampling facilitated successful exploration of an experience otherwise hidden from researchers. Despite the stigma of abortion, women talk with social contacts about abortion, and thus can connect each other via chain-referral sampling.

Most data about abortion in contexts where it is illegal are extrapolated from PAC data [20]. Our data highlight the extent to which such studies may underestimate how many abortions take place in a community, and potentially misestimate morbidity and mortality of abortions that do occur, although women who receive PAC do not necessarily represent only the least safe abortions, but also include women who had incomplete or worrisome abortions. Participants described keeping their abortions secret; perhaps many abortions would not have been reported in household surveys.

We found, as others have across sub- Saharan Africa, that women use local plants boiled down to concentrated tea to induce abortions [19]; several species have demonstrated uterine contractile activity [21]. Drawbacks to herbal methods include the inability of tea-makers to control pharmacoactive agent doses, the possibility of side-effects, and lack of data about efficacy and safety. Our participants often did not know what methods were used by health providers to terminate their pregnancies. Herbal methods may be more welcome for this reason—drinking a concocted tea may be less threatening than undergoing a procedure with unfamiliar methods. In mainland Tanzania and Zanzibar, women turn to traditional birth attendants and pharmaceutical retailers for help with abortions, as they offer greater convenience, privacy, and lower costs than physicians [22, 23]. Finally, while a distinction does exist between an abortion (illegal) and PAC (legal), women may not see this distinction, especially in contexts where both the providers, and methods of abortion and PAC may be the same.

While many participants noted using drinks made from local plants, multiple participants used strong black tea alone or in conjunction with local plants. We believe that we are the first to report induced abortion via ‘strong black tea:’ participants described boiling down up to 1 kilogram of black tea leaves to make one cup of tea which they drank. Tea with very high caffeine content, such as one cup made from 1 kg of tea leaves, could theoretically cause abortion. A meta-analysis of 43,000 pregnancies found a small but statistically significant [OR 1.36 (95 % confidence interval 1.29–1.45)] increase in spontaneous abortions among women consuming more than 150 mg caffeine daily [24]; the average regular cup of tea contains approximately 50 mg of caffeine, and 1 kg of tea leaves makes approximately 400 cups, leading to 20,000 mg of caffeine in a cup made from 1 kg of tea leaves. In other work, Klebanoff et al. [25] assessed serum levels of paraxanthine, a caffeine metabolite, to estimate caffeine doses at matched time points in women who did and did not have spontaneous abortions. Extremely high serum paraxanthine concentration levels were associated with spontaneous abortion [25]. Perhaps the concentrated black tea used by our participants contains enough caffeine to induce abortions. While only one woman successfully terminated her pregnancy using *only* strong black tea, the women who used black tea in combination with other herbs considered the black tea to be an active part of the treatment necessary to terminate their pregnancies. No women talked about the black tea as an inert vehicle for whatever else they boiled along with the black tea leaves. We submit that black tea as an abortion method is important because if a woman believes she terminated a pregnancy having used black tea (solely or in combination with other items), that woman contributes to the dialogue that circulates among Zanzibari women regarding how to terminate a pregnancy with this method.

While we cannot preclude the possibility that some women may have used ineffective methods to attempt induced abortion, and may instead have had spontaneous abortions or delayed menses, we demonstrate that multiple methods of abortion are known and used with the intent of terminating unwanted pregnancies in Zanzibar. Several more dangerous abortion methods described in other studies and from PAC providers we interviewed—including inserting objects into the vagina or uterus—were not mentioned by our participants [22, 26–27] (S. Yoseph, A. Gossa, and E. Tadesse, “A survey of illegal abortion in Addis Ababa, Ethiopia,” unpublished, 1993). It is particularly noteworthy that participants described the presence of both medical/herbal and surgical abortion, and that they found a range of non-traumatic options available.

### Limitations

Our study benefited from chain-referral sampling to understand hidden behaviors among women in Zanzibar. Our methodology, however, favored participation from women who lived near, or were willing to travel to, the urban center where the study was based. Our small sample size precluded use of statistical adjustments to produce generalizable samples and using chain-referral does not allow for true characterization of the sample in reference to the larger population. Our sample is not generalizable to Zanzibar as a whole, but is reflective of women living close to an urban center.

Chain-referral sampling may have been more likely to capture women who had recent abortions and/or more complicated abortions, as these may have been remembered more by their acquaintances. Alternatively, women with complicated abortions may have been *less* likely to want to participate. By its design, our study only captured women who told someone about their abortion; we cannot comment on the experiences of women who were able and/or wanted to keep their terminations more secret. Finally, women with unsuccessful terminations and women who died were not captured with our design. Because we recruited only women who did terminate a pregnancy, our study design excludes women who tried but failed to terminate a pregnancy. This may be a large number, and represents a dual public health problem: continuation of unwanted pregnancy *and* potential sequelae from ineffective abortion attempts.

Some participants described long past abortions, which may lead to recall bias regarding the details of their termination experience or whether or what sort of contraception they were using at the time they became pregnant. Gestational age at the time of termination was by participant self-report, and there is no way to confirm the validity of the dating. Additionally, many women were uncertain of the specifics regarding the methods of their terminations, as mentioned above.

## Conclusion

The surest way to prevent unsafe abortion is to prevent unwanted pregnancies. In our study, although the women did not want to become pregnant at the time they conceived, only 16 % of women reported using modern contraception at that time. Given the participants’ reasons for abortion, it is evident contraception promotion is needed. The majority of our participants were students or women with young children; focusing contraception access efforts to these groups at high risk for unintended pregnancies should be considered. While increasing contraceptive use will not eliminate the need for abortion, it will reduce the number of unintended pregnancies.

Social norms about abortion’s immorality, abortion’s illegal status, and widespread beliefs about abortion’s health risks present particular challenges for improving abortion care in Zanzibar. Insofar as women believe that abortion is necessary for some reasons, this rationale may serve as an entry point for lawmakers to advocate for changed laws, and for health care workers to provide abortions in cases permitted by law.

Misoprostol for abortion was not widely known in Zanzibar in 2010; no participants reported using misoprostol to terminate their pregnancies. However, at that time, health care providers were receiving training on using misoprostol for PAC, and we expect that this knowledge, as well as understanding about misoprostol from mainland Tanzania, may have brought the use of misoprostol to Zanzibari women. Many women in our study obtained successful abortions from medical providers, demonstrating that some providers give abortion care, even in a legally restricted environment. This context–providers knowledgeable in performing terminations and the likely introduction of misoprostol–indicate that Zanzibar would be a suitable place for a public health risk reduction strategy to prevent maternal deaths associated with unsafe abortion, such as was employed by the Uruguayan Ministry of Health [28]. Concurrent expansion of PAC services – including community awareness of their availability – and revisions of legal policies surrounding abortion would facilitate making ‘safer’ unsafe abortions truly safe, such that there are trained abortion providers with appropriate oversight.

## Abbreviations

PAC: post abortion care
RA: research assistant
